# The role of lipopolysaccharides in diabetic retinopathy

**DOI:** 10.1186/s12886-022-02296-z

**Published:** 2022-02-22

**Authors:** Xinran Qin, Haidong Zou

**Affiliations:** 1 Department of Ophthalmology, Shanghai General Hospital, Shanghai Jiao Tong University School of Medicine, Shanghai, China; 2 Shanghai Eye Diseases Prevention & Treatment Center, Shanghai Eye Hospital, Shanghai, China; 3 Shanghai Engineering Center for Precise Diagnosis and Treatment of Eye Diseases, Shanghai, China; 4 National Clinical Research Center for Eye Diseases, Shanghai, China; 5 Shanghai Key Laboratory of Fundus Diseases, Shanghai, China

**Keywords:** Lipopolysaccharides, Diabetic retinopathy, Gut leakage, Dysbiosis, Inflammation, Blood-retina barrier, Retinal glia

## Abstract

Diabetes mellitus (DM) is a complex metabolic syndrome characterized by hyperglycemia. Diabetic retinopathy (DR) is the most common complication of DM and the leading cause of blindness in the working-age population of the Western world. Lipopolysaccharides (LPS) is an essential ingredient of the outer membrane of gram-negative bacteria, which induces systemic inflammatory responses and cellular apoptotic changes in the host. High-level serum LPS has been found in diabetic patients at the advanced stages, which is mainly due to gut leakage and dysbiosis. In this light, increasing evidence points to a strong correlation between systemic LPS challenge and the progression of DR. Although the underlying molecular mechanisms have not been fully elucidated yet, LPS-related pathobiological events in the retina may contribute to the exacerbation of vasculopathy and neurodegeneration in DR. In this review, we focus on the involvement of LPS in the progression of DR, with emphasis on the blood-retina barrier dysfunction and dysregulated glial activation. Eventually, we summarize the recent advances in the therapeutic strategies for antagonising LPS activity, which may be introduced to DR treatment with promising clinical value.

## Background

Diabetes mellitus (DM) is a chronic metabolic syndrome characterized by abnormally high blood glucose levels. Absolute or relative lack of insulin caused by pancreatic β-cell dysfunction, insulin resistance, or both is the main reason for hyperglycemia [1]. The prevalence of DM was approximately 460 million people worldwide in 2019, and it is expected to rise to 700 million by 2045 [2], which poses a significant threat to global health. Hyperglycaemia, dyslipidaemia, insulin resistance, and metabolic pathway dysregulation are considered the core pathophysiological mechanisms of DM, leading to a series of complications involving multiple organ functions [1]. With an in-depth understanding of DM, oxidative stress, immune abnormalities, genetics, and epigenetics are currently considered significant contributors to the development of DM and its complications [3]. Diabetic retinopathy (DR) is the principal ophthalmic complication of DM, and it is clinically categorised into non-proliferative DR (NPDR) and proliferative DR (PDR) based on ophthalmoscopically visible microangiopathies [4]. Approximately 35% of diabetic patients have different degrees of retinopathy, and nearly 10% of them can progress to blindness [5]. In the Western world, DR is the primary cause of blindness in the working-age population.

Lipopolysaccharides (LPS), also known as endotoxins, are a key component of the outer membrane of gram-negative bacteria released after lysis. LPS has an amphiphilic and tripartite structure comprising a highly variable O antigen, core oligosaccharide, and lipid A (the main virulence factor) [6]. As a vital pathogen-associated-molecule-pattern (PAMP) in gram-negative bacteria, LPS can induce an innate immune defence and trigger inflammatory cascades in the host [7]. Several studies have found an association between high-level serum LPS and the progression of diabetic microvascular complications [8], which suggests that LPS may participate in retinal pathology.

This review examines the clinical and preclinical evidence on the association between LPS and DR, explores the possible role of LPS in the progression of DR, and summarizes recent advances in potential strategies for antagonising LPS in DR treatment.

## Increased levels of serum LPS in diabetic patients

Accumulating clinical evidence has demonstrated elevated serum LPS in diabetic patients with or without overt infection, also known as metabolic endotoxemia [8]. Therefore, the source of serum LPS in diabetic patients deserves further examination.

Gut microbiota-derived LPS should be of primary consideration. Gut microbiota is a group of 10^11^–10^12^ bacteria that colonise the human intestine and interact with the host throughout their lifespan. Its existence plays an essential role in maintaining intestinal homeostasis [9]. As a diverse ecosystem subjected to natural variations, the gut microbiota is highly flexible in different individuals and is implicated in numerous diseases, including DM [10, 11]. Significant shifts in the ratios of dominant phyla or outgrowth of pathobionts (symbiotic gut bacteria that may become pathogenic when appearing in large numbers) can result in a disease-prone status, referred to as dysbiosis [12, 13]. To date, sufficient studies have revealed marked differences in the gut microbiota composition of diabetic patients. A decline in microbial diversity and the growth of Firmicutes over Bacteroidetes are the principal features of the gut microbiome in diabetic patients [14]. The increased abundance of gram-negative opportunistic pathogens (some strains of *Bacteroides*, *Proteobacteria*, *Enterobacter,* and *Escherichia*) and decreased abundance of short-chain fatty acid (SCFA)-producing bacteria (such as *Fecalibacterium prausnitzii*, *Eubacterium rectale*, and some species in *Roseburia and Lachnospira*) are also universal traits in diabetic patients and experimental animals [15–17]. The contributing factors of dysbiosis in DM warrant a more detailed discussion. Currently, the mainstream belief attributes dysbiosis to four sources, including nutrients, host immunity, intestinal mucosa, and medication [12]. For example, high-fat and low-fibre diets in type II diabetes are often associated with increased *Proteobacteria* in the gut, bile acid pool, circulating LPS, and decreased levels of SCFA-producing bacteria [18–20]. The standard anti-hyperglycemic drug, metformin, increases *Escherichia coli* in the gut [21]. Furthermore, diabetic patients with DR have a significant imbalance in anti-endotoxin immunity during the disease course. The highest concentration of serum anti-LPS-IgA was detected in patients with NPDR that had macular oedema, aneurysms, haemorrhages, and portions of solid exudate; in addition to vascular changes, much lower concentrations of anti-LPS-IgA were identified in PDR, and the lowest concentrations of antiendotoxin antibodies were found in patients with PDR complicated by neovascular glaucoma [22].

**Fig. 1:**
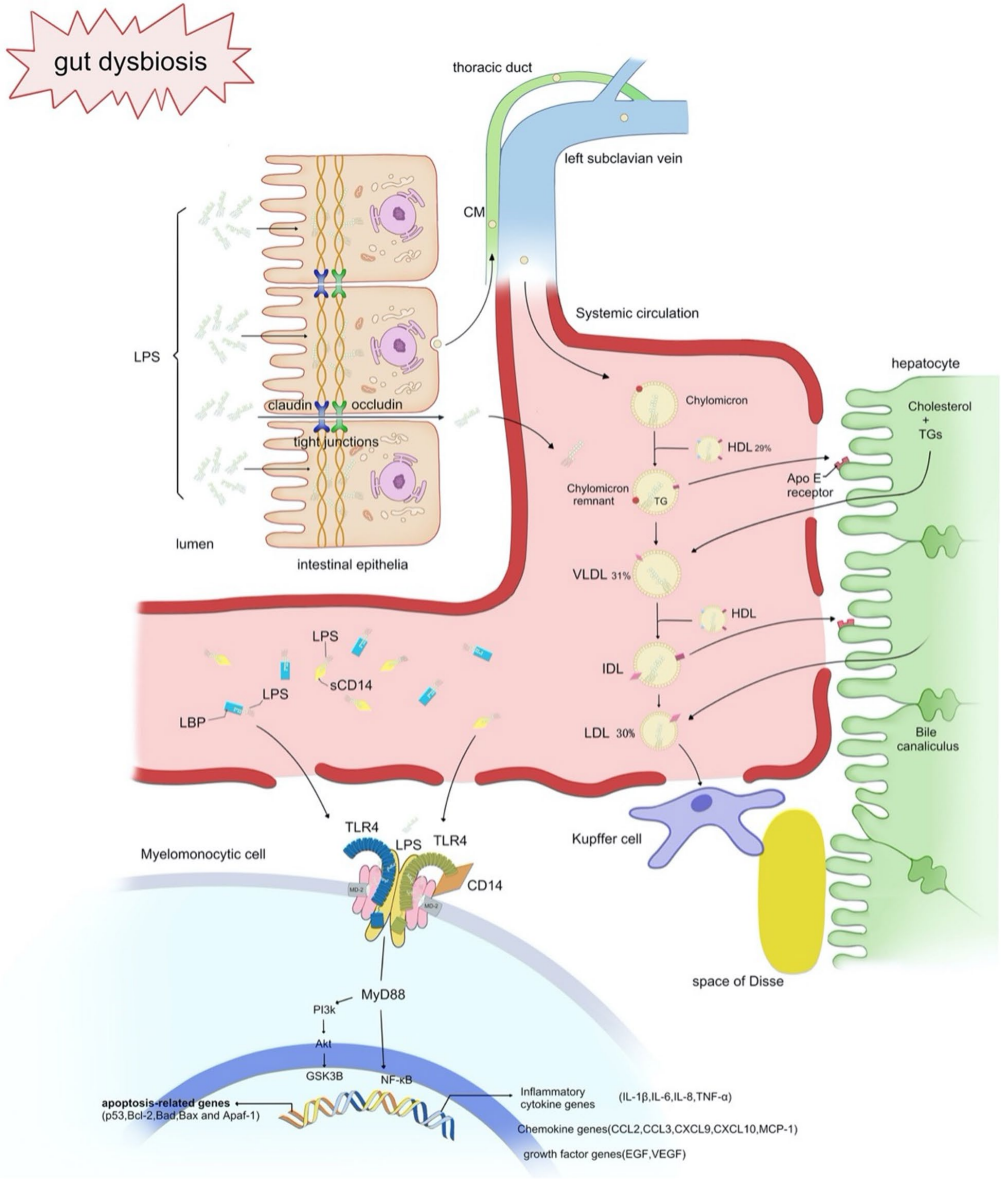
Schematic illustration of the pathogenesis of endotoxemia in diabetes mellitus, depicting the processes of translocation from the intestinal epithelial cells, transport in the circulation, and subsequent elimination by hepatic Kupffer cells, as well as cellular signaling in response to LPS at a molecular level

Metabolic endotoxemia can be aggravated by gut leakage in DM. Generally, tight junctions between intestinal epithelial cells (IECs) form an effective barrier for the healthy to resist the translocation of gut bacteria and their components into the bloodstream. Nevertheless, the extensively identified gut barrier dysfunction in diabetic patients may provide an opportunity for bacterial products to enter the bloodstream and elicit deleterious systemic effects [23, 24]. Previous studies have widely hypothesized that hyperglycemia is the culprit of gut leakage in diabetic patients through glucose transporter 2-dependent transcriptional reprogramming of IECs [25]. With substantial exploration of dysbiosis, its role in the increased gut permeability is gaining attention. For example, the decrease in *Bifidobacterium* can lead to a deficiency of glucagon-like peptide-2, which downregulates the expression of tight junction proteins between IECs [26], and the increase in some invasive strains of *Escherichia* may elicit direct damage to the IECs [27], while the decrease in SCFAs impairs the barrier function by influencing adenosine 5’-monophosphate-activated protein kinase activity and the assembly of tight junctions [17, 28]. Collectively, gut leakage and dysbiosis in diabetic patients allow an increased number of LPS to enter the bloodstream. Circulating LPS can spontaneously self-aggregate owing to its amphiphilic nature [5]. The aggregates quickly bind to LPS-binding protein (LBP) in the serum, and LBP can catalytically transfer them to soluble cell-differentiation 14 (sCD14) [29, 30]. Unique in their interactions with LPS, LBP and sCD14 can be designated soluble pathogen-recognition receptors and indicators of LPS activity. Ultimately, 90% of serum LPS can be captured by liver macrophages (i.e., Kupffer cells) within 1 h and inactivated by acyloxyacyl hydroxylase. However, the impaired Kupffer cell function in diabetic patients dampens the clearance of LPS [31], leading to the persistence of high-level serum LPS. (Fig. 1).

## Endotoxemia and the progression of DR are closely related

For decades, DR has been regarded as a microvascular disease, with advances in diagnostic technology, however, DR manifests early signs of neurofunctional alterations even before the appearance of vascular pathology [32]. Accordingly, the comprehensive definition of DR includes retinal vasculopathy and neuropathology, highlighting its complex and dynamic pathogenesis.

Hyperglycemia has long been hypothesized as the central pathophysiology of DR that causes four classical metabolic abnormalities in the retina: the activation of protein kinase C and the hexosamine pathway, the polyol pathway flux, and the production of advanced glycation end products (AGEs) [33]. The downstream effects mainly include the osmotic damage to retinal capillaries, oxidative stress in multiple retinal cells, and increased expression of vascular endothelial growth factor (VEGF) [34]. Specifically, AGE accumulation has been considered to play an indispensable role in the pathogenesis of DR and correlate with disease severity. At the molecular level, AGEs can damage the function and structure of various proteins by nonenzymatic crosslinks, especially antioxidant enzymes and collagens, which are detrimental to retinal capillary tonicity [35]. In addition, the interaction with its receptor contributes to the erroneous activation of multiple proinflammatory and proangiogenic pathways [36]. Of note, a recent study reported the clinical use of skin autofluorescence to assess AGE accumulation, which can serve as a non-invasive and reliable biomarker for identifying the patients at risk of sight-threatening DR [37].

Growing evidence has revealed the chronic low-grade inflammatory nature of DR. Elevated concentrations of inflammatory cytokines, such as interleukin (IL)-1β, IL-6, IL-8, tumour necrosis factor α (TNF-α), chemoattractant protein-1 (MCP-1), and VEGF, have been detected in the ocular tissues (vitreous and/or aqueous humour) of diabetic patients at different stages of DR [38–41]. Additionally, increasing numbers of anti-inflammatory drugs exhibit beneficial outcomes for DR treatment. For example, intravitreal steroids (such as Ozurdex and Iluvein) are approved to handle DR patients with severe complications [42, 43], and clinical trials to determine the efficacy of IL-6 inhibitors, such as EBI-031(clinicaltrials.gov ID: NCT02842541) and tocilizumab (clinicaltrials.gov ID: NCT02511067), have been carried out. Furthermore, approved drugs (such as aflibercept, bevacizumab and ranibizumab) that are extensively used in clinical practice can also work through inflammatory pathways of the retina [44].

The retina has long been regarded as an immune-privileged organ, as it is physically isolated from peripheral pathogens by the blood-retina barrier (BRB) [45]. BRB is a multicellular structure that can be divided into two layers: The inner layer is composed of tight junctions between the retinal endothelial cells (RECs) resting on the basement membrane, which is covered by astrocytes, Müller cells, and pericytes, that form the retinal neurovascular unit (NVU) [46]. The intactness of the NVU is a prerequisite for vascular integrity and for effectively preventing the passage of macromolecules across the barrier. The outer layer consists of tight junctions between retinal pigment epithelial cells (RPE), which is responsible for the separation of choroidal vasculature from neuroretina and permits the flux of specific molecules into the retina to regulate the dynamic balance of retinal metabolism [47]. Based on the current understanding of the fundamental role of inflammation in the pathogenesis of DR, high serum LPS in DM may be a significant candidate contributor to DR progression. Accumulating evidence suggests that periphery LPS have access to the eye and are sufficient to induce innate immune responses in the retinal cells, with or without BRB dysfunction, and there do exist traces that systemic LPS challenge may be associated with the progression of DR.

First, there may be high concentrations of LPS in the diabetic eyes. A previous study identified significantly increased levels of LBP and sCD14 in the vitreous humour of PDR patients, which indirectly demonstrated an increase in the intraocular LPS in DR [48]. Several studies have also found an increase in the level of sCD14 in the aqueous humour of patients with diabetic macular oedema (DME), a sight-threatening complication of DR. The elevated concentration of sCD14 is associated with that of VEGF, which is crucial for the development of DR. Combined with the analysis of optical coherence tomography images, it was also found that the higher sCD14 levels in diffuse DME patients closely correlated with the increase in the number of optically highly reflective foci in the retina, suggesting increased severity of retinal inflammation [49]. Despite the lack of direct evidence on the existence of LPS in diabetic eyes, a localization study confirmed the ocular distribution of LPS following intravenous injection in rabbits [50]. This may have resulted from the circular openings of approximately 800 Å in diameter on the choroidal capillary wall [51].

Second, peripheral LPS can induce and aggravate the retinal pathology. Repeated intraperitoneal injections of low-dose LPS gave rise to pathological manifestations mimicking DR in healthy mice, including activation, proliferation, and migration of retinal microglia; infiltration of the retina by monocyte-derived macrophages; and the highly reproducible breakdown of the BRB accompanied by subretinal fluid accumulation [52]. In parallel, systemic administration of low-dose LPS caused a 3.5-fold increase in endothelial cell injury and thinning (10-13 µm) of the posterior retina in diabetic mice [53], further deterioration of neural function, augmented loss of photoreceptors, worsening of synaptic connectivity, and an increased number of activated microglia in P23H rats (a retinal neurodegenerative animal model) [37–39, 54].

## Possible mechanisms of LPS in the pathogenesis of DR

### General pathogenic mechanisms of LPS

LPS is a classical exogenous pro-inflammatory and pro-apoptotic mediator that requires CD14 and Toll-like receptor 4 (TLR4) to elicit cellular responses. Given the lack of transmembrane and intracellular regions, CD14 expressed on the surface of myelomonocytic cells monomerizes LPS aggregates and presents them to the TLR4–myeloid differentiation factor 2 (MD2) complex to transduce signals [55]. In the case of the complex, the N-terminal and central domains of TLR4 provide the charge that is complementary for MD2, subsequently forming a stable heterodimer for specific binding to LPS. With further internalisation, the TLR4–MD2 complex initiates the myeloid differentiation primary response protein 88 (MyD88)—dependent pathway, leading to the activation of multiple transcription factors, especially nuclear factor-κB (NF-κB) and interferon regulatory factor 3, and the upregulation of the expression of various inflammatory mediators, including pro-inflammatory cytokines (such as IL-1β, IL-6, IL-8 and TNF-α), chemokines [C–C motif ligand 2 (CCL2), CCL3, C-X-C motif ligand 3 (CXCL3), CXCL9, CXCL10 and MCP-1], growth factors [epidermal growth factor, platelet-derived growth factor (PDGF), and VEGF], and enzymes for vasoactive substances [inducible nitrous oxide synthase (iNOS) and cyclooxygenase-2 (COX2)]. Besides, AIM2 (absent in melanoma 2)-like receptors and nucleotide-binding oligomerization domain-like receptors (NLRs) are also pivotal receptors of LPS that participate in inflammasome assembly. Inflammasomes interact with the CARD (caspase activation and recruitment domain) of the adaptor protein ASC (apoptosis-related dot-like protein containing CARD) and hydrolyse procaspase-1, thereby recruiting and activating caspase-1. Activated caspase-1 further hydrolyses pro-IL-1β and pro-IL-18 to intensify inflammation [56]. Of note, TLRs and NLRs are widely expressed on retinal cells, and pharmacological blockade of these receptors strongly ameliorates the retinal pathology induced by LPS [57, 58].

LPS is also an essential mediator of apoptosis. Recent studies have revealed that intracellular LPS can activate mouse caspase-11 (corresponding to human caspase-4/5) precursors to initiate the process of apoptosis in an atypical manner. Intracellular LPS can spontaneously aggregate into a micelle structure. The negatively charged polar heads of the micelles that locally reach a critical concentration (10–20 µg/mL) can interact with the positively charged residues in caspase-4/5/11, resulting in the oligomerization of the caspase [59]. In addition, the pro-inflammatory cytokines, glutamate, reactive oxygen species (ROS), and nitride oxide (NO) produced by LPS-stimulation can accumulate in the microenvironment of the injured sites, leading to the sequential activation of caspase [60]. Moreover, LPS-TLR4 signalling can upregulate the expression of other pro-apoptotic genes by interacting with the PI3K/AKT/GSK3β and AMPK/GSK3β-Nrf2 pathways [61, 62].

### LPS participates in retinal neurodegeneration

#### Microglial activation

Microglia are the predominant immune cells responsible for surveillance and are the first responders of the innate immune system in the eye. When the surrounding environment is disturbed by pathogenic stimuli, microglia can immediately transform into amoeba-like forms, also called pro-inflammatory phenotypes (M1), induce low-grade inflammation to defence, and demonstrate strong capacities for proliferation, migration and phagocytosis [63]. With the elimination of the stressors, they restore the transcriptome under surveillance or transform into an anti-inflammatory phenotype (M2), which can release anti-inflammatory cytokines (such as IL-4, IL-10, IL-13, and transforming growth factor) to maintain balance. Nevertheless, the disrupted balance of microglial polarisation has been identified in the retina of diabetic patients at early stages, rendering it a hallmark of DR pathogenesis [64].

Persistent stimulation with LPS may reduce the plasticity of the microglial transcriptome in diabetic eyes, leading to excessive M1 polarisation. This was initially demonstrated by the morphological and phenotypic changes in microglia and a significant increase in inflammatory mediator secretion when activated by LPS [65]. In addition to the canonical LPS-TLR4-MyD88 pathway, LPS may contribute to retinal inflammation by inducing necroptosis in microglia. Necroptosis is a form of cell death that is mediated by receptor-interacting protein kinase 1/3 (RIPK1/3). TLR4 activation is a key initiation step in necroptosis. LPS-activated TLR4 recruits the cytoplasmic adaptor protein TIR-domain containing adaptor inducing interferon-β through the endosomal platform. With further interaction between RIPK3 and RIPK1, they act on the effector protein, mixed-lineage kinase like domain, which undergoes a configurational change and translocates to the plasma membrane to increase permeability, leading to necroptosis of microglia with a marked release of inflammatory mediators that, consequently, intensifies LPS-induced retinal inflammation [66].

Microglial activation orchestrates neurodegeneration and vasculopathy in the retina of DR. Activated microglia construct an extensively interconnected internet that contributes to cell death in various retinal cells. First, the cytokines released by LPS-activated microglia can trigger the activation of retinal neural glia and elicit neuroinflammatory damage [60]. This cytokine cocktail has been proven neurotoxic, leading to a marked loss of retinal neurons. Moreover, activated microglia with phagocytosed rhodopsin-positive particles were substantially detected in the photoreceptor layer of patients with retinal degenerative diseases [67], suggesting deleterious effects on photoreceptor cell death. Microglial activation also contributes to retinal vascular damage due to its regulatory role in the retinal vasculature. In vitro experiments have shown that activated microglia can promote the angiogenic process and vascular permeability of co-cultured RECs through the secretion of VEGF and PDGF [68]. When clearing vascular exudates and cell debris, they also penetrate the basement membrane of the inner BRB to engulf some of the normal RECs leading to an increase in the number of acellular capillaries and resultant vascular leakage. The interaction between pericytes and microglia has been recently uncovered. The upregulation of NADPH oxidase subunits and downregulation of uncoupling protein 2 expressions are found in pericytes co-cultured with LPS-activated microglia, which leads to increased ROS production and secretion of pro-inflammatory mediators (such as iNOS and TNFα) in pericytes, negatively influencing BRB maintenance [69].

#### Reactive gliosis is an indispensable ingredient of neurodegeneration

Retinal neural glia are neuron-supportive cells with significant functions in immune modulation, metabolism regulation, and nourishment of neurones, which are essential for the homeostasis of the neuroretina network [70]. Specifically, Müller cells are the most widely distributed in the retina, accounting for 90% of the retinal glia. They form a radial supporting structure along the entire width of the retina, which is critical for metabolism and intercellular communication. Comparatively, astrocytes are confined to the neuroretina, and this distribution is closely related to the presence of retinal vasculature [71]. More importantly, astrocytes provide energy substrates for neurones and secrete neurotrophic factors and antioxidants to promote neural survival [72]. Reactive gliosis refers to the proliferation and activation of Müller cells and astrocytes in response to retinal stress, which includes processes of neuroinflammation, phagocytosis of apoptotic neurones and cell debris, and secretion of neurotrophic factors [73]. The upregulated expression of the glial fibrillary acidic protein (GFAP) is an early sign of reactive gliosis, and increased GFAP expression can be detected in the aqueous humour of NPDR patients, the retina of DR mice, and the LPS-treated glial cells in vitro [74–76], this suggests the pivotal role of gliosis in the progression of DR. Under physiological conditions, a delicate balance is maintained between the damaging and protective effects of reactive gliosis [73]. Long-term exposure to LPS, however, may give rise to chronic gliosis and result in the disturbance of this balance, namely, damage far surpassing protection. Eventually, the inability for activated glia to maintain appropriate support for BRB and the neuroretina could lead to the exacerbation of neurodegeneration in DR.

In Müller cells, the transcriptomic analysis showed alterations in 78 genes in the 6th month of the course of DR, one-third of these genes was under the regulation of LPS-TLR4 signalling, such as VEGF, intercellular cell adhesion molecule-1 (ICAM-1), IL-1β, IL -6, MCP-2, NO, and COX2, which are closely related to neuroinflammation [77]. Further, mass spectrometric analysis of Müller cells in response to LPS revealed an increase in the proteins ascribed to antigen-presenting cells (APCs) and function to interact with T-cells [78], suggesting that activated Müller cells may act as noncanonical APCs to participate in the neuroinflammation underlying DR. There is also a program of bidirectional microglia-Müller cell signalling that can mediate adaptive responses within the retina following initial microglial activation [79]. The response of Müller cells may augment initial retinal inflammation and guide the intraretinal mobilisation of microglia through chemokines and cell adhesion. LPS is also a key regulator of hepcidin expression in Müller cells through TLR4-dependent transcriptional reprogramming. The upregulation of hepcidin and subsequent downregulation of ferroportin are associated with increased oxidative stress and apoptosis within the retina in vivo, and chronic exposure to LPS may disrupt iron homeostasis and retinal function [80]. Moreover, LPS-activated Müller cells can exhibit decreased expression and incorrect positioning of potassium channels (Kir4.1), combined with subtle changes in aquaporin, which results in a disturbance of water and potassium transport at the capillary-Müller cell interface and leads to retinal oedema and violation of the NVU function [81]. In astrocytes, LPS can increase the A1-type profile (marked by increased C3 expression, namely reactive astrocytes) by activating JAK2/STAT3 signalling. A1-type astrocytes can produce multiple inflammatory mediators to aggravate neuroinflammation [82]. It is worth noting that astrocytes require the presence of activated microglia to effectively respond to LPS stimulation in vitro [83], which emphasizes the sequential activation of retinal glia.

### LPS exacerbate retinal vasculature damage and BRB dysfunction

As the core component of the outer BRB, RPE can transform into a pro-inflammatory state in response to LPS [84], which express high levels of cytokine receptors (including IL-1R,-6R, -8RA, IFNAR1, IFNGR1/2) and secrete a range of inflammatory mediators (including IL-6, -8, -17, -18, IFN-γ, MCP-1, and VEGF) to induce morphological damage to tight junctions. IL-6 and IL-8 mediate LPS toxicity through an autocrine feedback loop, which leads to RPE degeneration and further exacerbation of the outer BRB disintegration. As demonstrated in a proteomic analysis of RPE in response to LPS, there is a significant downregulation of proteins related to mitochondrial respiration and cell cycle checkpoints along with an upregulation of proteins related to lipid metabolism, amino acid metabolism, cell–matrix adhesion, and endoplasmic reticulum (ER) stress [85]. The nucleotide-binding domain, leucine-rich repeat-containing family, and pyrin domain-containing 3 (NLRP3) is excessively expressed in RPE treated with LPS in vitro [86]. Furthermore, elevated levels of IL-1β, NLRP3, ASC, and caspase-1, along with increased GFAP, Iba-1 (a marker of activated microglia), pro-inflammatory cytokines (TNF-α and IL-6) and pro-angiogenic markers (ICAM-1 and VEGF) were identified in a PDR mouse model, suggesting that the NLRP3 inflammasome plays a pivotal role in the advanced stages of DR. Paradoxically, LPS-treated RPE indicates classical phospholipases D activation that modulates the autophagic process and serves as a protection mechanism [87]. Low-grade activation by systemic LPS administration leads to a preconditioning effect that transiently improves the function and structure of RPE [88]. These protective effects, at least in part, may explain the minor role of endotoxemia in retinal pathology at the very early stage of DM. Long-term exposure to LPS and pro-inflammatory cytokines, however, may induce chronic inflammation that is overwhelmingly detrimental RPE viability, barrier properties and phagocytosis function, thereby contributing to destructive changes in the retinal environment of DR.

**Fig. 2:**
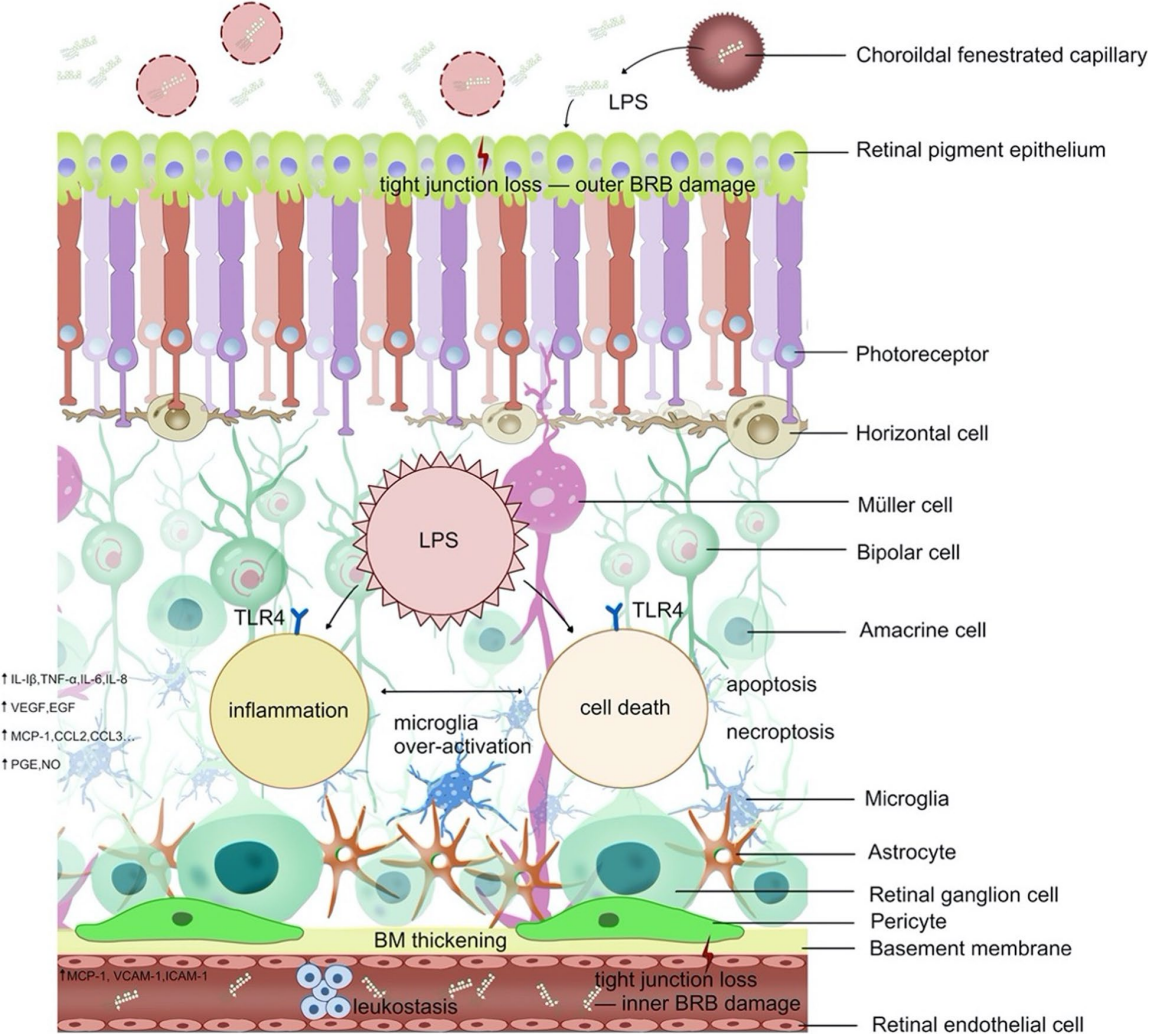
Depiction of the pathobiological events of LPS in the human retina, depicting the interactions of LPS with retinal cells and LPS-triggered pathological manifestations in DR

Similar inflammatory responses are induced by LPS in the inner BRB. Being extremely susceptible to pro-inflammatory cytokines, RECs not only become the primary victim of retinal inflammation, but also increase the expressions of chemokines (such as CCL8, CXCL10, and MCP-1) and intracellular adhesion molecules (such as ICAM-1, VCAM-1, and P-selectin), leading to a process referred to as leukostasis [89]. Leukostasis means that peripheral leukocytes begin to marginate, roll, firmly adhere to REC, and migrate into the retina ultimately. This adhesive interaction between leukocytes and RECs is associated with endothelial swelling, an increase in the number of acellular capillaries, and other microangiopathies [90]. Chronic ER stress is another LPS-triggered pathophysiological event in RECs, which results in apoptosis and contributes to retinal degeneration [91]. On the other hand, the damage signals of ongoing retinal inflammation can recruit peripheral macrophages, T-lymphocytes and dendritic cells to migrate into the retina through the leaky blood vessel wall. Subsequently, macrophages that infiltrate the retina can spontaneously undergo transcriptional reprogramming via the TLR4-dependent pathway, expressing the same surface molecules as microglia and performing similar pro-inflammatory and pro-angiogenic functions to amplify retinal neurovascular injury [92]. The accumulation of immune cells on the surface or within the lumen of retinal vasculature leads to the mechanical occlusion of the retinal vasculature and an increase in non-perfusion areas, which further augments the hypoxic and ischaemic damage to the retina of DR and even induces neovascularization [93, 94] (Fig. 2).

## Anti-LPS strategies in DR therapeutics

The treatment of DR is still challenging, as it involves multiple interplaying pathogenic mechanisms. Photocoagulation laser treatment and intravitreal injection of anti-VEGF agents are significant milestones in the history of DR therapy [95, 96]. Although both have achieved clinical success through inhibiting neovascularization, there are obvious limitations, such as being effective only for advanced patients with severe neurodegeneration and requiring repeated surgical procedures [97]. Therefore, novel strategies that cover a wide range of mechanisms are urgently needed. Based on the previously discussed role of LPS in the pathogenesis of DR, interventions specifically antagonising LPS may be promising for DR treatment, which warrants further research.

### Agents to modulate the gut microbiome and reconstruct barrier integrity

Probiotics are a class of microorganisms beneficial for regulating the host mucosal immunity, promoting the balance of the gut microbiota, improving the gut barrier function, and maintaining nutrient metabolism [98]. At present, the long-lasting benefits of probiotics on the modulation of the gut microbiota and recovery of gut barrier function in diabetic patients have gained attention [99]. Before formal introduction into clinical practice, large-scale clinical trials are required to determine the long-term efficacy.

In both animal models and clinical trials, orally taking probiotic strains, such as *Lactobacillus rhamnosus, Lactobacillus gasseri, Lactobacillus casei, Lactobacillus acidophilus, Streptococcus thermophilus, Lactobacillus bulgaricus,* and *Bifidobacterium lactis*, for 5–12 weeks exhibited positive effects on DM, including reducing blood glucose, serum LPS, and pro-inflammatory cytokines (TNF-α, IL-6, and IL-β) levels. Furthermore, a combination of different probiotic strains exhibits even broader benefits on the management of diabetic patients [100–102]. The majority of existing studies merely focused on its value in DM treatment. *Yamazaki* et al. recent work, however, did suggest that *Lactobacillus paracasei* KW3110 protects RPE against premature senescence and aberrant expression of tight junction proteins caused by chronic inflammatory stress in vitro and improves the grade of eye fatigue in healthy subjects [103], which strongly suggests the potential of probiotics in chronic eye disorders including DR.

### Agents to ameliorate the activity of circulating LPS

Antimicrobial peptides (AMP), an essential ingredient of innate immunity, are synthesized and secreted by immune cells in response to PAMP signals to eliminate pathogens by direct killing effects and immune regulation [104]. AMP can selectively destroy the integrity of bacterial cell membranes, inhibit the synthesis of bioactive macromolecules (such as nucleic acids, proteins, and enzymes), recruit and activate innate immune cells. Defensins are one of the most important groups of AMPs and can be divided into three subfamilies, α, β, and θ, according to their disulfide bond positions [105]. Compelling evidence has confirmed that human β defensin can reduce LPS-induced expression of TNF-α and IL-6 in both mouse and human macrophages [106, 107]. Compared to natural AMPs, the newly developed synthetic anti-LPS peptide (SALP) has less cytotoxicity and stronger ability to neutralize LPS toxicity. For example, peptide 19–2.5 can bind firmly to LPS and transform the lipid A into an inactive form, thus impairing LPS activity [108]. Notably, it can also interact with many types of antibiotics and reduce serum TNF-α levels. In an animal septic model, the peptide 19–2.5 successfully lowered the incidence of septic cardiomyopathy and prevented heart failure [109].

Current anti-infection treatment of diabetic patients is facing difficulties. For one thing, the abuse of antibiotics can lead to the emergence of multi-drug resistant bacteria, which poses a devastating threat to handling severe infections at the advanced stages of DM [110]. For another, LPS released by the killed bacteria can be a source of endotoxemia. In contrast, AMP combined with minimal-dose antibiotics can effectively treat the endotoxemia in DM by direct killing and neutralizing LPS toxicity, and help to avoid the antibiotics abuse, which may further become a potential intervention for DR management.

### Agents to regulate retinal glial reactivity to LPS

Glial activation is now considered central to the development of DR [72], so modulating their reactivity to LPS may be helpful to alleviate the LPS-induced retinal inflammation. Olfactory ensheathing cells (OECs), a type of glial cells, can secrete various cytokines involved in immune regulation and neuroinflammation. *Xie* et al. found that retinal OEC grafts can promote the conversion of microglia from a pro-inflammatory phenotype to an anti-inflammatory phenotype through the JAK-STAT3 pathway, thereby exhibiting anti-inflammatory and neuroprotective capabilities [111]. *Jha* et al. reported the neuroprotective role of adipose tissue-derived mesenchymal stem cells (ASC-CCMs), a kind of pluripotent stem cells, in the central nervous system inflammatory diseases. Upon LPS stimulation, ASC-CCMs release a variety of bioactive molecules, such as extracellular superoxide dismutase, immunomodulatory proteins (such as indoleamine 2,3-dioxygenase), and TNF-stimulated gene 6 protein (TSG-6). Among them, TSG-6 can increase the expression of anti-inflammatory mediators in a STAT3-dependent manner, thereby promoting the phenotypic transformation of retinal microglia [112]. Besides, *Xian* et al. have shown the ability of mesenchymal stem cell-derived exosomes (MSC-Exos) to combat LPS-induced astrocyte reactivity via the Nrf2-NF-κB signalling pathway in vivo [113]. Due to the merit of crossing the BRB, exosome has been currently regarded as an efficient drug delivery tool for use in retinopathy. Upon MSC-Exos treatment in this study, the LPS-upregulated expression of pro-inflammatory cytokines can return to normal. Related calcium signal abnormalities and mitochondrial dysfunction were ameliorated, which closely correlate with retinal neurodegeneration. Accordingly, these cell therapies may be beneficial in ameliorating retinal inflammation with promising neuroprotective function, however, clinical trials are still required to determine the clinical value.

**Fig. 3:**
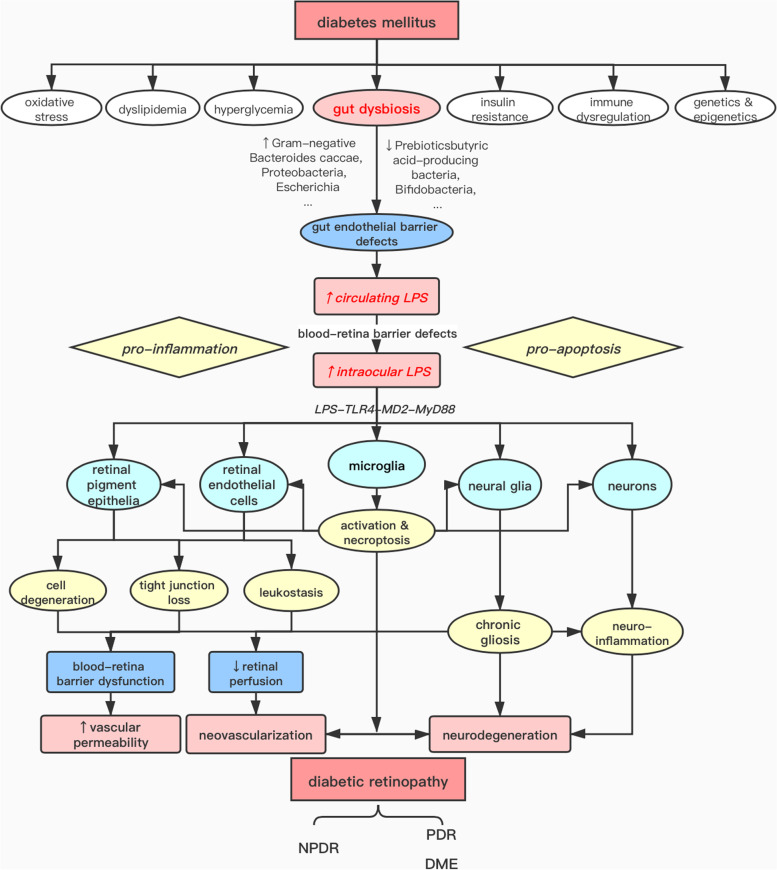
Graphic overview of the role of LPS in the progression of diabetic retinopathy, summarizing the sequential events from endotoxemia derived from gut leakage and dysbiosis, the increase in the activity of intraocular LPS, the responses to LPS in each type of retinal cells, to the consequent retinal pathology in DR

Ultimately, microRNA (miRNA), a member of non-coding RNA, has emerged as a pivotal regulator in microglial activation. MicroRNA can cause mRNA silencing or degradation by binding to mRNA, thereby downregulating the expression of its target genes [114]. Currently, increasing numbers of studies have focused on the regulatory role of miRNA in retinal chronic inflammatory conditions. First, a set of dysregulated miRNAs (miR-20a-5p, miR-20a-3p, miR-20b, miR-106a-5p, miR-27a-5p, miR-27b-3p, miR-206-3p, and miR-381-3p) are identified in serum and retinas of diabetic mice [115]. They can modify the expression of VEGF, brain-derived neurotrophic factor, and cAMP response element-binding protein 1, before the occurrence of vasculopathy. In addition, miR-21, miR-223, miR-204, miR-30a, miR-34a, and miR93 also contribute to downregulating microglia activation in vivo or in vitro, and delivery of those miRNAs exhibits neuroprotective effects in the degenerative or ageing retina [116–120]. On the contrary, miR-155 and miR-146a are reported to upregulate inflammatory and apoptotic pathways in microglia, thereby targeting these miRNAs may ameliorate inflammatory response in the degenerative retina [121–123]. With the assistance of nanotechnology and adeno-associated viral vector-based strategy, RNA technology has become an attainable therapeutic approach that can improve retinal drug availability. For example, *Amadio* et al. reported the use of nanocarriers complexed with small interfering RNA silencing Human antigen R (HuR) in DR rats. This treatment demonstrated potent retinal protection by significantly dampening the expression of retinal HuR and its target VEGF [124]. Collectively, regulating the miRNA network may contribute to restraining the retinal inflammation induced by LPS in DR patients, leading to better neuroprotection in DR management. (Fig. 3).

## Conclusions

This review summarizes the clinical and preclinical research on the association between LPS and DR, suggesting a significant role of LPS in the progression of DR. Sufficient studies have found that endotoxemia is common in diabetic patients [7, 8], and that gut dysbiosis and defective epithelial barrier may be the culprits. Peripheral LPS have access to the eye through the openings of choroid blood vessels [50], allowing endotoxemia to involve in the pathogenesis of DR. However, this effect is restrained by relatively low LPS activity and intact host immunity at the very early stages of DM. In contrast, there is persistently high-level serum LPS combined with impaired immunity at the advanced stages [22], thus circulating LPS can play a significant part in the progression of DR. By disturbing ocular homeostasis, intruding LPS activate a wide variety of retinal cells to intensify the retinal inflammatory cascades as well as cell degeneration [57, 64, 80–88], which manifests as aggravation of BRB dysfunction, reduction of retinal blood perfusion, and deterioration of neural dysfunction.

There are great limitations in the current treatment of DR [97]. Given the complexity in retinal structure, multiple pathogenic mechanisms interplay in DR, and nearly every cell component could contribute to the development of DR. Therefore, a single target often has compromised benefits. As the pivotal role of endotoxemia in the pathogenesis of DR has been gradually elucidated, LPS may become a potential target for DR therapeutics. The strategies include reconstruction of the gut microbiota through probiotics, neutralization of the activity of serum LPS by AMPs/SALPs, and regulation of the retinal glial responsiveness to LPS, which are worthy of further research. Overall, the association between LPS and DR reveals the existence of the gut-retina axis, and an in-depth understanding of this axis will not only help to elucidate the more comprehensive pathogenic mechanisms of DR and other retinopathies such as age-related macular degeneration and retinopathy of prematurity, but it also sheds lights on better therapeutics. 

## Data Availability

Not applicable

## Abbreviations

AGE: Advanced glycation end product
APC: Antigen-presenting cell
BRB: Blood-retina barrier
CXCL: C-X-C motif ligand
DM: Diabetes mellitus
DME: Diabetic macular oedema
DR: Diabetic retinopathy
ER: Endoplasmic reticulum
GFAP: Glial fibrillary acidic protein
ICAM: Intercellular cell adhesion molecule
IECs: Intestinal epithelial cells
IL: Interleukin
LBP: Lipopolysaccharide-binding protein
LPS: Lipopolysaccharides
MCP: Chemoattractant protein
MD: Myeloid differentiation factor
NLR: Nucleotide-binding oligomerization domain-like receptor
NVU: Neurovascular unit
NPDR: Non-proliferative diabetic retinopathy
PAMP: Pathogen-associated-molecule-pattern
PDR: Proliferative diabetic retinopathy
REC: Retinal endothelial cell
RIPK: Receptor-interacting protein kinase
RPE: Retinal pigment epithelium
sCD: Soluble cell-differentiation 14
TLR4: Toll-like receptor
TNF-α: Tumour necrosis factor α
VEGF: Vascular endothelial growth factor
